# Outcomes and Challenges of Flap Reconstruction for Pressure Injuries in Clinically Complex Patients

**DOI:** 10.3390/jcm15124814

**Published:** 2026-06-21

**Authors:** Stephanie M. Mueller, Ovya Ganesan, Ana M. Pachano-Bravo, Harriet Kiwanuka, LaYow C. Yu, Joanna Woodman, Erin Bertagnolli, Dennis P. Orgill

**Affiliations:** 1 Division of Plastic and Reconstructive Surgery, Brigham and Women’s Hospital, 75 Francis St., Boston, MA 02115, USA; smmueller@mgh.harvard.edu (S.M.M.); hkiwanuka@bwh.harvard.edu (H.K.); 2 Harvard Medical School, 25 Shattuck St., Boston, MA 02115, USA; apachanob@gmail.com (A.M.P.-B.); layow_yu@hms.harvard.edu (L.C.Y.); 3 Dartmouth Geisel School of Medicine, 1 Rope Ferry Road, Hanover, NH 03755, USA; ovya.ganesan.med@dartmouth.edu; 4 Wound Care Center, Brigham and Women’s Hospital, 75 Francis St., Boston, MA 02115, USA; jwoodman1@bwh.harvard.edu (J.W.); bnolli13@gmail.com (E.B.)

**Keywords:** pressure injury, pressure ulcer, flap reconstruction, reconstructive surgery, spinal cord injury

## Abstract

**Background**: Pressure injuries (PIs) are common in patients with limited mobility and may require flap reconstruction for definitive management. However, postoperative complications and PI recurrence frequently occur. Certain flap types may be more prone to poor outcomes. This study evaluated outcomes after flap reconstruction for PIs in a medically complex population. **Methods**: We performed a retrospective review of patients who underwent flap reconstruction for sacral, ischial, trochanteric, or lateral malleolar PIs by a single surgeon at a tertiary care center between 2015 and 2023. Patient demographics, comorbidities, neurologic status, wound characteristics, flap type, and postoperative outcomes were collected. Outcomes were analyzed at the flap level. **Results**: Sixty-eight patients underwent 101 flap reconstructions. Most patients were male (68%), and spinal cord injury was present in 71%. Medical comorbidity burden was high, including anemia (61%), malnutrition (42%), preoperative osteomyelitis (44%), stool exposure near the wound (49%), and near-universal urinary incontinence. Postoperative complications were common across flap types, most commonly wound dehiscence and PI recurrence. New PIs developed at non-operative sites in about 14% of reconstructions during recovery. During the eight-year follow-up period, 19 (28%) patients expired and 21% of reconstructions were complicated by recurrence at the operative site. **Conclusions**: Flap reconstruction remains an important treatment for advanced PIs but is associated with high complication and recurrence rates in patients with substantial comorbidities and limited mobility. These findings support careful patient selection, preoperative optimization, and multidisciplinary postoperative care focused on preventing new PIs.

## 1. Introduction

Pressure injuries (PIs) consist of damage to the skin and underlying tissues caused by prolonged pressure and shearing forces. They are one of the most common conditions affecting acutely hospitalized and long-term residential care patients. It is estimated that 2.5 million PIs are treated in acute care facilities every year in the United States, with the majority of these occurring prior to hospital admission [1,2]. PIs serve as a source of major psychological and physiological burden to affected individuals and have been associated with reduced quality of life and increased stress [3].

The development of PIs is multifactorial. They typically develop over bony prominences, where sustained external pressure and compression between bone and soft tissue impair perfusion, resulting in local ischemia and hypoxia [4]. Shearing forces cause architectural distortion of the skin and tissue, further exacerbating damage in the presence of pressure [5]. If pressure over muscle and soft tissue is not relieved within two to three hours, prolonged ischemia can lead to irreversible tissue injury and progression through a predictable cascade of inflammation, cellular death, and tissue necrosis extending from the deep tissues to the skin [6,7,8]. Additional risk factors include reduced vascular supply, sensory loss, malnutrition, and impaired ability to perform pressure-relieving maneuvers [9].

PI management focuses on both preventing further tissue injury and promoting wound healing. The mainstay of treatment for PIs consists of individualized repositioning plans and high-quality wound care [1,10]. Advanced PIs that fail conservative management may require flap reconstruction to provide durable soft tissue coverage, reduce the risk of chronic infection and osteomyelitis, and address complications associated with longstanding nonhealing wounds, including Marjolin ulcer formation, an aggressive cutaneous squamous cell carcinoma that may arise after decades of chronic tissue injury [4,5,7]. Flap reconstruction involves transferring well vascularized skin, soft tissue, fascia, and sometimes muscle to the affected region [11]. This provides substantial coverage and oxygen supply to the injured tissue, allowing for proper healing [12]. Flap reconstruction is typically reserved for patients with substantial life expectancies who will benefit the most from the associated decreased morbidity. Such patients are typically those with spinal cord injuries given their severe mobility limitations and relatively young age [13].

Although flap reconstruction can provide durable soft tissue coverage and improve patient outcomes, it is also associated with a substantial risk of postoperative complications. These complications include surgical site infection, wound dehiscence, flap necrosis, hematoma, and PI recurrence. The reported rate of wound dehiscence following flap reconstruction for PI according to the ACS NSQIP is 2.4%, while the reported wound dehiscence rate ranges from 10 to 31% in retrospective cohort studies [13,14,15,16,17,18]. In such retrospective cohort studies, PI recurrence rates also range from 1.4 to 31.3% [13,15,16,18]. These complications may prolong wound healing, necessitate additional procedures or hospitalizations, and negatively affect functional recovery. In particular, recurrence remains a significant challenge, as it can result in repeated cycles of wound breakdown, prolonged dependence on caregivers, reduced quality of life, increased healthcare utilization, and failure to achieve durable reconstruction.

Prior studies have sought to identify patient- and procedure-related factors that contribute to these adverse outcomes. Patient risk factors include younger age, reduced mobility/dependent functional status, low serum albumin, body mass index (BMI) < 18.5, active smoking status, scoliosis, and diabetes mellitus [13,14,15,16,17]. Additionally, lack of social support and knowledge of proper wound care can lead to these complications [13]. The literature also reports factors related to flap reconstruction that increase complication rates, such as presence of multiple PIs, large PIs, long operative times, need for perioperative blood transfusion, and use of thigh V-Y flaps and fasciocutaneous gluteal rotational flaps [13,14,16,18].

Due to the variability of complication rates across studies, further characterization of outcomes following different flap types is warranted. Additionally, one outcome that is not discussed in the literature is the development of PIs at new locations during the postoperative recovery period. We sought to better characterize this finding as a reflection of the substantial physical and caregiving demands associated with recovery after flap reconstruction. Given the high burden of comorbidities, immobility, and long-term care needs in patients undergoing reconstruction for advanced PIs, improved characterization of postoperative outcomes may help guide flap selection, risk stratification, patient counseling, and perioperative management.

## 2. Materials and Methods

### 2.1. Study Design and Patient Selection

We conducted a retrospective review of electronic medical records (EMR) for patients who underwent flap reconstruction for PIs performed by a single plastic surgeon (DPO) at a tertiary academic center between January 2015 and December 2023. This study was approved by the Institutional Review Board of Brigham and Women’s Hospital (protocol number 2023P000380). Patients were identified by retrospectively reviewing a single surgeon’s list of patients who underwent flap reconstruction for PIs. Eligible patients included those who received a fasciocutaneous or musculocutaneous flap or local tissue arrangement (LTR) for sacral, ischial, trochanteric, or lateral malleolar PIs. Patients were evaluated for surgical candidacy prior to reconstruction. Relative contraindications to flap reconstruction included active systemic infection, severe malnutrition (serum albumin < 3.0 g/dL), active nicotine use, uncontrolled diabetes mellitus, inability to tolerate general anesthesia, inability to comply with postoperative pressure offloading requirements, and inadequate social support for postoperative care. When feasible, modifiable risk factors were optimized prior to surgery. Patients managed with surgical debridement alone, negative pressure wound therapy (NPWT) without flap reconstruction, or other closure techniques were excluded. Other exclusion criteria included age under 18 years old, missing data, and follow-up less than six weeks.

### 2.2. Data Collection

A single reviewer collected data on patient sociodemographic characteristics, comorbidities, pre-operative medications, PI characteristics, surgical details, and postoperative outcomes from the EMR. To reduce data abstraction errors and improve accuracy, each chart underwent two independent rounds of review by the same reviewer. Sociodemographic characteristics included sex, age, race, and BMI. Comorbidities were recorded based on diagnosis lists and preoperative clinic notes, with a focus on cardiopulmonary disease, diabetes mellitus, neurological impairment, psychiatric conditions, and the presence of stool exposure or urinary incontinence. Anemia was defined as hemoglobin <13.0 g/dL in males and <12.0 g/dL in females, malnutrition as serum albumin < 3.5 g/dL, and renal insufficiency as estimated glomerular filtration rate (eGFR) < 60 mL/min/1.73 m^2^. Tobacco or nicotine use was recorded if cessation was required prior to surgery. Surgical data included PI location, stage, flap type, and presence of preoperative soft-tissue infection and osteomyelitis. PIs were staged according to the National Pressure Injury Advisory Panel (NPIAP) staging system. Osteomyelitis was defined based on histopathologic findings from intraoperative bone biopsy specimens obtained at the time of PI excision. Perioperative antibiotic use was obtained from the discharge summary. Of note, length of stay and nutritional support variables were not collected as part of the study.

### 2.3. Outcomes

Six-week postoperative complications included wound dehiscence, hematoma, readmission for surgical complications, and new PI formation, defined as development of a PI at a site distinct from the operative reconstruction site. Long-term outcomes included mortality and PI recurrence, defined as a new PI at the operative site occurring more than eight weeks after surgery. Adequate short-term follow-up was defined as six weeks and was obtained through postoperative clinic notes in the EMR. Adequate long-term follow-up was defined as six months and obtained through the EMR or telephone follow-up. Mortality was obtained by the EMR and public obituaries.

### 2.4. Operative Technique

Patients are positioned prone with careful padding of bony prominences and protection of the orbits. For trochanteric pressure injuries, patients are positioned in the lateral decubitus position. The ulcer is stained with methylene blue, infiltrated with local anesthetic containing epinephrine, and completely excised. Surgical debridement is performed according to previously described techniques, with complete excision of nonviable soft tissue and infected bone as indicated [19]. An ostectomy is performed, and the resected bone is sent for pathologic evaluation. A bone biopsy is subsequently obtained from bone that does not appear grossly infected and is submitted for microbiologic analysis to guide antibiotic therapy.

Flap selection is individualized to provide adequate dead-space obliteration and wound closure while preserving future reconstructive options in the event of recurrence. The principles guiding flap selection and reconstructive planning have been described in detail previously [19]. One or two closed-suction drains are placed. Incisions are covered with abdominal pads. Standard perioperative antibiotic prophylaxis is administered and continued until culture results are available. Most patients are co-managed by Infectious Disease specialists for antibiotic selection and duration.

### 2.5. Postoperative Care

Patients are maintained on a strict no-sitting protocol for six weeks postoperatively. Those who are non-ambulatory are on strict bedrest and are repositioned every two hours to minimize pressure on the flap reconstruction. Ambulatory patients may get out of bed for standing and walking. Patients are discharged only after a definitive antibiotic plan has been established and adequate home support services or rehabilitation placement have been arranged to ensure continued adherence to pressure offloading and repositioning requirements. Patients use pressure-reducing air mattresses to prevent new PIs. Drains are maintained until output is less than 30 mL over a 24 h period. Dressings are changed every few days.

Patients are seen for follow-up six-weeks postoperatively. During this visit, Wound Center nurses and physician assistants remove sutures and assess patients for postoperative complications, including wound-related complications and the development of new PIs. If there are no complications, patients may begin a progressive sitting protocol. They are seen monthly until healing is complete, or more frequently if there are complications.

### 2.6. Statistical Analysis

Descriptive statistics were used to summarize patient characteristics. Categorical variables were presented as absolute frequencies (n) and percentages (%) and were compared using the chi-square test. Continuous variables were described as mean ± standard deviation (SD) or median with interquartile range (IQR), according to the data distribution, and were compared using analysis of variance (ANOVA) or the Kruskal–Wallis test, as appropriate. Statistical significance set at *p* < 0.05. Statistical analysis was performed using R software, version 4.4.2 (R Foundation for Statistical Computing, Vienna, Austria).

This study was conducted and reported in accordance with the Strengthening the Reporting of Observational Studies in Epidemiology (STROBE) guidelines.

## 3. Results

### 3.1. Cohort Demographics

A total of 73 patients were identified, with five excluded due to having less than six weeks of follow-up and missing data. The final cohort included 68 patients with at least six weeks of follow-up. Twenty-seven percent were younger than 40 years, 34% were aged between 40 and 59 years, and 39% were aged 60 years or older. Most patients were male (68%) and White (81%). Regarding BMI, 10% were underweight, 41% had a normal BMI, 32% were overweight, and 16% were obese. Baseline demographic and clinical characteristics of the study cohort are summarized in Table 1.

Hypertension (34%) and psychiatric conditions (46%) were common comorbidities. Anemia (61%) and malnutrition (42%) were particularly prevalent. Diabetes mellitus (21%) and asthma (16%) were observed, while chronic obstructive pulmonary disease (COPD) was uncommon (3%). Renal insufficiency (10%) and coagulopathy (9%) were also present. Preoperative osteomyelitis was identified in 44% of patients. Active smoking was rare (3%), and no patients had active liver failure, Parkinson’s disease, or a history of chemotherapy or cyclosporine use. Stool exposure to the wound was present in 49% of patients, while only 13% had a diverting ostomy at the time of reconstruction. Urinary incontinence was highly prevalent (94%). All PIs were stage IV at the time of surgery.

Spinal cord injury was present in 71% of patients, with 38% being quadriplegic and 52% paraplegic. Additional neurologic conditions included spina bifida (10%), demyelinating diseases (4.4%), cerebral palsy (2%), and spastic paresis (2%). Cognitive impairment, cerebrovascular accident, and traumatic brain injury were uncommon.

Among the 68 included patients, a total of 101 flap reconstructions were performed. Twenty patients received more than one flap during their procedure, whereas six patients received subsequent procedures for recurrent pressure injuries (*n* = 3), development of new pressure injuries at different anatomic sites (*n* = 2), or both (*n* = 1), totaling seven additional flaps. The interval between procedures ranged from one to six years. Subsequent reconstructions were performed on non-healing stage IV sacral and ischial PIs.

Gluteal flaps accounted for the majority (*n* = 76) and included gluteal fasciocutaneous, gluteus maximus, and gluteus musculocutaneous flaps. Local tissue rearrangement (LTR) flaps (*n* = 10) included W-plasty transposition, LTR advancement, and LTR procedures. Tensor fascia lata (TFL) flaps (*n* = 3) were used for standard trochanteric PI coverage. Miscellaneous flaps (*n* = 12) encompassed gracilis flaps, posterior thigh flaps, peroneal perforator flaps, triangular-type flaps, V-Y advancement flaps, skin advancement flaps, readvancement of gluteal fasciocutaneous flaps, TFL flaps used outside the trochanteric region, and other unspecified flaps.

To avoid overrepresentation of individual patients, only the first flap reconstruction for each patient was included in Table 2, which summarizes patient characteristics stratified by flap type. Age, sex, race, and BMI distribution were similar across groups (all *p* > 0.05). Corticosteroid use (*p* = 0.031), immunotherapy use (*p* = 0.034), COPD prevalence (*p* = 0.017), and anemia (*p* = 0.0022) differed by flap type. These differences were driven by very small subgroup sizes, particularly within the LTR and TFL groups, and should be interpreted cautiously.

### 3.2. Cohort Outcomes

Of the 101 flaps, wound dehiscence, hematoma, readmission, and new PI were monitored for six weeks. PI recurrence and mortality was monitored over the course of the eight-year study period. During the long-term study period, 19 patients expired, all more than one-year postoperatively. Seventeen patients had at least six months of follow-up documented in the EMR. An additional 48 patients were successfully contacted by telephone to obtain follow-up information. Three patients were unable to be reached through telephone call for long-term follow-up, leaving 65 patients (98 flaps) with at least six-months of follow-up who were included in the long-term outcomes assessment. Because some patients underwent multiple reconstructions, postoperative complications are reported at the flap level rather than the patient level.

Postoperative complication rates varied by flap type (Table 3). Among gluteal flaps, the rates of wound dehiscence, hematoma, readmission, and new PIs were 21%, 4.2%, 2.8%, and 16%, respectively. Among LTR flaps, wound dehiscence and new PI rates were 50% and 10%. TFL flaps showed no dehiscence, readmissions, or hematomas, but a 33% rate of a new PI. Miscellaneous flaps had dehiscence rates of 42%, with no readmissions, hematomas, or new PIs. The most common reason for readmission was a postoperative hematoma. For long-term complications, PI recurrence was most frequently seen in TFL flaps (67%) and gluteal flaps (24%). Mortality was demonstrated across flap types.

### 3.3. Case Presentations

**Figure 1:**
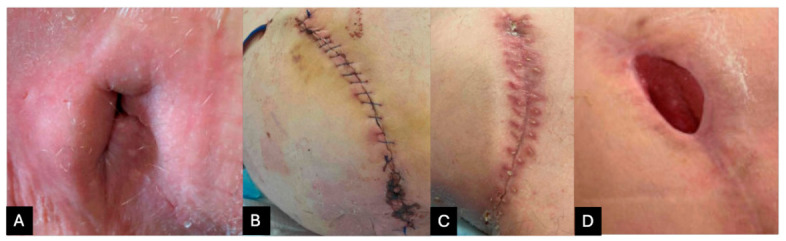
Gluteal flap with pressure injury recurrence. (**A**) 65-year-old male with paraplegia and stage IV right ischial pressure injury. (**B**) One week following wound excision, ostectomy, and gluteus maximus muscle flap closure. (**C**) Healing five-weeks postoperatively. (**D**) Recurrence of ischial pressure injury one-year postoperatively.

**Figure 2:**
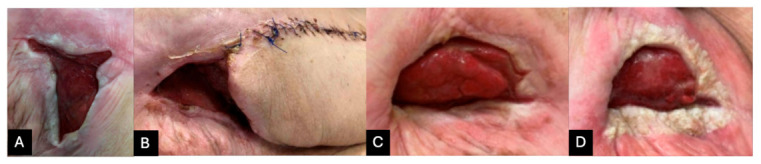
Local transposition flap with wound dehiscence. (**A**) 37-year-old male with paraplegia and stage IV right ischial pressure injury. (**B**) Wound dehiscence two-weeks following wound excision and local transposition flap closure. (**C**) Persistently open wound seven months postoperatively and (**D**) one year postoperatively.

**Figure 3:**
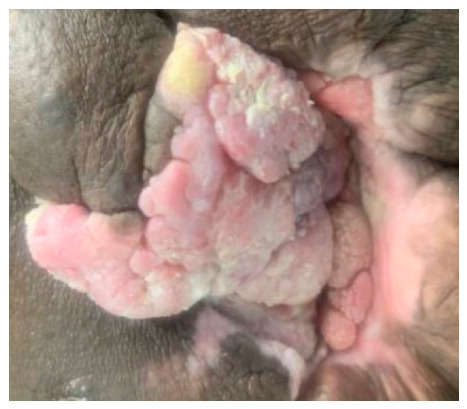
Marjolin ulcer: a 51-year-old male with paraplegia and a history of recurrent sacral pressure injuries despite two decades of wound care and multiple surgical interventions, including flap closure. The wound eventually progressed to a Marjolin ulcer, which was managed with immunotherapy, as radiation and surgery would complicate wound healing. This illustrative case was not part of the study cohort and is included to highlight a severe long-term complication of pressure injuries.

Representative cases are detailed below to demonstrate complications, such as wound dehisence, PI recurrence, and Marjolin ulcer formation (Figure 1, Figure 2 and Figure 3).

## 4. Discussion

In this retrospective cohort study of patients undergoing flap reconstruction for advanced PIs, we observed high rates of postoperative complications, including wound dehiscence, PI recurrence, and new PI formation at non-operative sites. These findings highlight the substantial physiologic and caregiving burden faced by this medically complex population and highlight the challenges of achieving durable wound healing despite surgical intervention.

### 4.1. Comparison with Prior Literature

The complication rates observed in our cohort are consistent with, and in some cases higher than, those reported in prior retrospective studies of flap reconstruction for PIs. Reported rates of wound dehiscence in the literature range from approximately 10–31%, while recurrence rates range from 1.4% to over 30%, depending on patient population, flap type, and follow-up duration [13,14,15,16,17,18]. In our study, wound dehiscence and recurrence remained common across flap types, reinforcing prior findings that flap reconstruction does not eliminate the risk of postoperative complications or recurrent disease.

Notably, our study provides additional insight by capturing the development of new PIs at non-operative sites during the postoperative recovery period, an outcome that has been inconsistently reported in prior studies. In our practice, patients undergoing reconstruction for pelvic PIs are restricted from sitting for at least six-weeks postoperatively to protect the flap. This recovery period can be physically and emotionally challenging, particularly for patients who rely on wheelchairs for mobility and independence in activities of daily living. Additionally, patients are limited in the positions they can safely maintain, as they must avoid both pressure on the flap site and prolonged pressure on other vulnerable areas.

The development of new PIs during recovery highlights the vulnerability of this population during prolonged immobilization and emphasizes that successful healing at the operative site does not necessarily translate to overall PI prevention. Prior work suggests that shorter immobilization protocols, such as four-week regimens, may allow earlier mobilization without increasing complication rates [20]. These findings warrant further investigation.

### 4.2. Patient Complexity, Malnutrition, and Medical Vulnerability

The high prevalence of anemia (61%) and malnutrition (42%) in our cohort highlights the medically fragile and nutritionally vulnerable nature of patients selected for flap reconstruction. Although laboratory predictors of healing were not formally analyzed in this study, prior work has demonstrated that nutritional status plays a critical role in flap success. In particular, serum albumin levels above 2.5 g/dL have been associated with improved outcomes following flap reconstruction for PIs [12]. Our findings emphasize the importance of careful preoperative optimization and patient selection in a population with significant baseline physiologic risk.

### 4.3. Immobility, Incontinence, and Wound Exposure

A defining feature of our cohort was the high prevalence of neurologic impairment and immobility, with 71% of patients having a spinal cord injury and the majority experiencing quadriplegia or paraplegia. These conditions inherently limit the ability to offload pressure and contribute to prolonged pressure over bony prominences. In addition, urinary incontinence was nearly universal across all flap types, and stool exposure to the wound was present in nearly half of patients, with relatively low rates of diverting ostomy at the time of reconstruction.

In our experience, many of our ambulatory patients are able to heal their PIs with high-quality wound care, particularly through technilogical advancements like negative pressure wound therapy (NPWT), and are able to avoid extensive surgery. However, our patients with neurologic impairments struggle to similarly heal their PIs.

Prior studies have demonstrated that immobility and incontinence are key contributors to PI development and impaired wound healing. Prolonged pressure and increased skin moisture accelerate tissue breakdown, while incontinence-associated dermatitis has been strongly linked to sacral PIs [21,22]. A non-randomized prospective study found that protective diverting colostomy was associated with increased healing rates and reduced infections following flap reconstruction for ischial and sacral PIs, emphasizing the importance of appropriate wound care and patient optimization [23]. Although we did not identify statistically significant associations between incontinence and specific postoperative complications, the near-universal presence of these risk factors in our cohort highlights the ongoing challenge of wound protection during recovery.

### 4.4. Multidisciplinary Care and Postoperative Management

Given the complexity of this patient population, our findings support the importance of a multidisciplinary approach to PI management before and after flap reconstruction. Multidisciplinary treatment protocols incorporating plastic surgery, wound care, nutrition, physical therapy, occupational therapy, and nursing have been shown to reduce complication and recurrence rates in patients with spinal cord injuries undergoing flap surgery. Nutritional support is particularly critical, as adequate protein and caloric intake are essential for wound healing. Physical therapy and structured rehabilitation protocols aim to optimize mobility while minimizing pressure-related injuries [17].

We also found that mental health services are an important component of wound healing, as psychiatric disease may further complicate recovery in patients with limited mobility. In our cohort, psychiatric comorbidities were common, with 46% of patients carrying a formal psychiatric diagnosis. We observed that these conditions often represented a substantial barrier to healing, as successful PI management requires consistent wound care, pressure offloading, and adherence to daily preventive measures. Patients with psychiatric disease may have greater difficulty maintaining these demanding care routines. Prior literature has similarly identified psychiatric disease as an independent risk factor for PI development in patients with spinal cord injuries [24,25]. These findings underscore the importance of integrating mental health support into multidisciplinary PI prevention and treatment strategies.

### 4.5. Mortality and Underlying Health Status

Although mortality was observed during long-term follow-up in 19 patients, death in this cohort was unlikely to be directly attributable to surgery, as all patients died more than 1 year postoperatively. Flap reconstruction is intended to reduce morbidity and improve quality of life by addressing chronic, nonhealing wounds that themselves carry substantial risk of infection and systemic complications. However, recurrent or new PIs, which are constant challenges for those with spinal cord injuries, may put patients at risk for such outcomes in the long term.

The literature also supports the high morbidity associated with spinal cord injuries. Prior studies have shown that infection is a leading cause of death in patients with spinal cord injury, even after controlling for PIs [26]. Additionally, this population faces a markedly increased risk of cardiovascular and metabolic disease, which further contributes to long-term mortality [27]. While the specific causes of death could not be reliably determined in our cohort, it is likely that chronic illness and systemic complications played a major role.

### 4.6. Strengths and Limitations

A key strength of this study is the extended follow-up period, which allowed assessment of long-term outcomes, particularly PI recurrence. Loss to longitudinal follow-up (three patients) and mortality during follow-up (19 patients) limited complete longitudinal outcome ascertainment.

This was a retrospective study conducted at a single center by a single surgeon, which may limit generalizability. The sample size, particularly within certain flap subgroups, was small, and statistically significant differences by flap type should be interpreted with caution. Additionally, laboratory predictors of healing and functional recovery milestones were not systematically analyzed.

### 4.7. Prevention and Future Directions

Despite advances in surgical reconstruction, this study demonstrates the ongoing difficulty of achieving durable healing in patients with advanced PIs. Ultimately, prevention remains the most effective strategy. Regular repositioning, vigilant skin monitoring, and early intervention are essential but may be difficult to implement in patients with limited mobility or insufficient social support.

Emerging technologies offer promising avenues for prevention. Telemedicine has been shown to reduce the incidence and severity of PIs in community-dwelling patients with spinal cord injuries through enhanced monitoring and early intervention [28]. Mobile health applications may further support PI prevention by enabling remote monitoring, risk assessment, patient education, and wound documentation [29,30]. Additionally, advances in pressure mapping systems and high-tech pressure-relieving mattresses may further reduce risk by optimizing pressure redistribution [31].

Future studies should prioritize prospective, multicenter designs with larger sample sizes to improve generalizability. Decision-support tools integrating medical, functional, and social factors represent a promising direction for improving patient selection and reducing recurrence [32].

Recently, Eddy et al. demonstrated that implementation of an interdisciplinary risk stratification tool significantly reduced one-year PI recurrence following flap surgery in veterans with spinal cord injuries [32]. While recurrence was common in our cohort, events were not confined to the first postoperative year, underscoring the need for longitudinal risk assessment frameworks that extend beyond early postoperative surveillance. Such tools align closely with the multifactorial vulnerabilities observed in our population and may help refine surgical candidacy and postoperative management.

## 5. Conclusions

Flap reconstruction remains an important treatment modality for advanced PIs but is associated with high rates of postoperative complications, recurrence, and development of new PIs in medically complex patients with limited mobility. These findings highlight the multifactorial challenges of achieving durable wound healing in this population and underscore the importance of careful patient selection, preoperative optimization, multidisciplinary postoperative care, and long-term PI prevention strategies. Future prospective studies are needed to improve risk stratification, optimize perioperative management, and reduce recurrence following reconstruction.

## Figures and Tables

**Table 1:** Baseline demographic and clinical characteristics of patients undergoing flap reconstruction for pressure injuries (*n* = 68). Abbreviations: eGFR, estimated glomerular filtration rate.

Characteristic	Overall (*n* = 68)
Age group, *n* (%)	
<40 years	18 (27%)
40–59 years	23 (34%)
≥60 years	26 (39%)
Sex	
Male	46 (68%)
Race, *n* (%)	
White	55 (81%)
Black	7 (10%)
Other	6 (8.8%)
Body mass index, *n* (%)	
Underweight	7 (10%)
Normal	28 (41%)
Overweight	22 (32%)
Obese	11 (16%)
Medical comorbidities *n* (%)	
Coronary artery disease	7 (10%)
Hypertension	23 (34%)
Asthma	11 (16%)
Chronic obstructive pulmonary disease	2 (2.9%)
Neurologic respiratory failure	3 (4.4%)
Diabetes mellitus	14 (21%)
Renal insufficiency (eGFR < 60)	7 (10%)
Malnutrition	22 (42%)
Anemia	41 (61%)
Coagulopathy	6 (8.8%)
Corticosteroid use	1 (1.5%)
Immunotherapy use	4 (5.9%)
Preoperative osteomyelitis	30 (44%)
Neurologic status, *n* (%)	
Quadriplegia	26 (38%)
Paraplegia	35 (52%)
Spinal cord injury	48 (71%)
Spina bifida	7 (10%)
Demyelinating disease	3 (4.4%)
Traumatic brain injury	2 (2.9%)
Cerebral palsy	1 (1.5%)
Spastic paresis	1 (1.5%)
Cerebrovascular accident	1 (1.5%)
Wound-related factors, *n* (%)	
Diverting ostomy	9 (13%)
Stool exposure to wound	33 (49%)
Urinary incontinence or catheter	64 (94%)
Other factors, *n* (%)	
Active smoking	2 (3.0%)
Psychiatric condition	31 (46%)
Radiation to operative site	2 (2.9%)

**Table 2:** Baseline patient characteristics stratified by flap type, including gluteal flaps, local tissue rearrangement, tensor fascia lata, and miscellaneous flaps. Abbreviations: SD, standard deviation; COPD, chronic obstructive pulmonary disease. In the case when a patient had more than one flap, only their first procedure is listed to avoid over counting comorbidities. * Indicates statistical significance.

Characteristic	Gluteal Flap (*n* = 56)	Local Tissue Rearrangement (*n* = 3)	Tensor Fascia Lata (*n* = 2)	Miscellaneous (*n* = 7)	*p* Value
Age group, *n* (%)					0.91
<40 years	16 (29%)	1 (33%)	0 (0%)	1 (14%)	
40–59 years	19 (35%)	1 (33%)	1 (50%)	2 (29%)	
≥60 years	20 (36%)	1 (33%)	1 (50%)	4 (57%)	
Sex					0.39
Male	39 (70%)	2 (67%)	2 (100%)	3 (43%)	
Race, *n* (%)					0.54
White	46 (82%)	3 (100%)	2 (100%)	4 (57%)	
Black	6 (11%)	0 (0%)	0 (0%)	1 (14%)	
Other	4 (7.1%)	0 (0%)	0 (0%)	2 (29%)	
Body mass index, *n* (%)					0.51
Underweight	7 (13%)	0 (0%)	0 (0%)	0 (0%)	
Normal	22 (39%)	2 (67%)	0 (0%)	4 (57%)	
Overweight	18 (32%)	1 (33%)	2 (100%)	1 (14%)	
Obese	9 (16%)	0 (0%)	0 (0%)	2 (29%)	
Medical comorbidities, *n* (%)					
Antithrombotic use, mean (SD)	0.34 (0.48)	0.00 (0.00)	0.50 (0.71)	0.29 (0.49)	0.62
Corticosteroid use	0 (0%)	0 (0%)	0 (0%)	1 (14%)	0.031 *
Immunotherapy use	2 (3.6%)	0 (0%)	1 (50%)	1 (14%)	0.034 *
Preoperative osteomyelitis	26 (46%)	2 (67%)	0 (0%)	2 (29%)	0.39
Hypertension	18 (32%)	2 (67%)	1 (50%)	2 (29%)	0.61
Asthma	8 (14%)	1 (33%)	0 (0%)	2 (29%)	0.58
COPD	1 (1.8%)	1 (33%)	0 (0%)	0 (0%)	0.017 *
Diabetes mellitus	14 (25%)	0 (0%)	0 (0%)	0 (0%)	0.29
Anemia	37 (67%)	1 (33%)	2 (100%)	1 (14%)	0.022 *
Malnutrition	17 (40%)	1 (50%)	2 (100%)	2 (40%)	0.40
Neurologic status, *n* (%)					
Spinal cord injury	39 (70%)	3 (100%)	2 (100%)	4 (57%)	0.44
Quadriplegia	23 (41%)	1 (33%)	1 (50%)	1 (14%)	0.56
Paraplegia	27 (48%)	2 (67%)	1 (50%)	5 (71%)	0.65
Wound-related factors, *n* (%)					
Diverting ostomy	7 (13%)	1 (33%)	1 (50%)	0	0.21
Stool exposure to wound	30 (54%)	0 (0%)	1 (50%)	2 (29%)	0.21
Urinary incontinence or catheter	52 (93%)	3 (100%)	2 (100%)	7 (100%)	0.82
Other factors, *n* (%)					
Active smoking	2 (3.6%)	0 (0.0%)	0 (0.0%)	0 (0.0%)	0.93
Psychiatric condition	28 (50%)	2 (67%)	0 (0.0%)	1 (14%)	0.14
Radiation to operative site	2 (3.6%)	0 (0.0%)	0 (0.0%)	0 (0.0%)	0.93

**Table 3:** Short-term complications (wound dehiscence, hematoma, readmission, new pressure injury) and long-term complications (pressure injury recurrence and mortality) stratified by flap type.

Complication	Gluteal Flap	Local Tissue Rearrangement	Tensor Fascia Lata	Miscellaneous	Total
Short-term complications *n* (%)	*n* = 76	*n* = 10	*n* = 3	*n* = 12	*n* = 101
Wound dehiscence	16 (21%)	5 (50%)	0 (0.0%)	5 (42%)	26 (26%)
Hematoma	3 (4.2%)	0 (0.0%)	0 (0.0%)	0 (0.0%)	3 (3.0%)
Readmission	2 (2.8%)	0 (0.0%)	0 (0.0%)	0 (0.0%)	2 (2.0%)
New pressure injury	12 (16%)	1 (10%)	1 (33%)	0 (0.0%)	14 (14%)
Long-term complications *n* (%)	*n* = 74	*n* = 10	*n* = 3	*n* = 11	*n* = 98
Pressure injury recurrence	18 (24%)	1 (10%)	2 (67%)	0 (0.0%)	21 (21%)
Mortality	14 (19%)	1 (10%)	1 (33%)	3 (27%)	19 (19%)

## Data Availability

The data presented in this study are not publicly available due to patient privacy concerns and institutional restrictions.

## Abbreviations

The following abbreviations are used in this manuscript, listed in alphabetical order:

BMI	Body mass index.
COPD	Chronic obstructive pulmonary disease.
eGFR	Estimated glomerular filtration rate.
EMR	Electronic medical record.
LTR	Local tissue rearrangement.
NPWT	Negative pressure wound therapy.
PI	Pressure injury.
SD	Standard deviation.
STROBE	Strengthening the Reporting of Observational Studies in Epidemiology.
TFL	Tensor fascia lata.
